# Development of Chronic Pain Conditions Among Women in the Military Health System

**DOI:** 10.1001/jamanetworkopen.2024.20393

**Published:** 2024-07-05

**Authors:** Andrew J. Schoenfeld, Madison N. Cirillo, Jonathan Gong, Matthew R. Bryan, Amanda Banaag, Joel S. Weissman, Tracey P. Koehlmoos

**Affiliations:** 1 Center for Surgery and Public Health, Department of Orthopaedic Surgery, Brigham and Women’s Hospital, Harvard Medical School, Boston, Massachusetts; 2 Henry M. Jackson Foundation for the Advancement of Military Medicine, Bethesda, Maryland; 3 Department of Orthopaedic Surgery, Brigham and Women’s Hospital, Harvard Medical School, Boston, Massachusetts; 4 Harvard Medical School, Boston, Massachusetts; 5 Center for Surgery and Public Health, Department of Surgery, Brigham and Women’s Hospital, Harvard Medical School, Boston, Massachusetts; 6 Center for Health Services Research, Uniformed Services University of the Health Sciences, Bethesda, Maryland

## Abstract

**Importance:**

The incidence of chronic pain has been increasing over the last decades and may be associated with the stress of deployment in active-duty servicewomen (ADSW) as well as women civilian dependents whose spouse or partner served on active duty.

**Objective:**

To assess incidence of chronic pain among active-duty servicewomen and women civilian dependents with service during 2006 to 2013 compared with incidence among like individuals at a time of reduced combat exposure and deployment intensity (2014-2020).

**Design, Setting, and Participants:**

This cohort study used claims data from the Military Health System data repository to identify ADSW and dependents who were diagnosed with chronic pain. The incidence of chronic pain among individuals associated with service during 2006 to 2013 was compared with 2014 to 2020 incidence. Data were analyzed from September 2023 to April 2024.

**Main Outcomes and Measures:**

The primary outcome was the diagnosis of chronic pain. Multivariable logistic regression analyses were used to adjust for confounding, and secondary analyses were performed to account for interactions between time period and proxies for socioeconomic status and combat exposure.

**Results:**

A total of 3 473 401 individuals (median [IQR] age, 29.0 [22.0-46.0] years) were included, with 644 478 ADSW (18.6%). Compared with ADSW in 2014 to 2020, ADSW in 2006 to 2013 had significantly increased odds of chronic pain (odds ratio [OR], 1.53; 95% CI, 1.48-1.58). The odds of chronic pain among dependents in 2006 to 2013 was also significantly higher compared with dependents from 2014 to 2020 (OR, 1.96; 95% CI, 1.93-1.99). The proxy for socioeconomic status was significantly associated with an increased odds of chronic pain (2006-2013 junior enlisted ADSWs: OR, 1.95; 95% CI, 1.83-2.09; 2006-2013 junior enlisted dependents: OR, 3.05; 95% CI, 2.87-3.25).

**Conclusions and Relevance:**

This cohort study found significant increases in the diagnosis of chronic pain among ADSW and civilian dependents affiliated with the military during a period of heightened deployment intensity (2006-2013). The effects of disparate support structures, coping strategies, stress regulation, and exposure to military sexual trauma may apply to both women veterans and civilian dependents.

## Key Points

**Question:**

Did the incidence of chronic pain among active-duty servicewomen (ADSW) and women civilian dependents differ between 2006 to 2013, a period of heightened combat and deployment intensity, and 2014 to 2020, a period of reduced combat intensity?

**Findings:**

This cohort study including 3 473 401 ADSW and dependents identified significant differences in the diagnosis of chronic pain among ADSW and dependents in 2006 to 2013 compared with 2014 to 2020. Chronic pain was documented in 14.8% of ADSW in service during 2006 to 2013 and in 11.3% of dependents from this period, compared with 7.1% in ADSW and 3.7% of dependents from 2014 to 2020.

**Meaning:**

This cohort study found strong and pervasive signals for the association of combat exposure with the subsequent diagnosis of chronic pain.

## Teaser

This cohort study assesses incidence of chronic pain among active-duty servicewomen and women civilian dependents associated with service during 2006 to 2013 compared with among like individuals at a time of reduced combat exposure and deployment intensity (2014-2020).

## Introduction

Chronic pain is a condition whose prevalence is increasing worldwide.^1^ In the US, chronic pain is one of the leading causes of disability in the working-age population, affecting as many as 100 million individuals.^2^ The treatment of chronic pain and its ramifications, including opioid dependence, addiction, substance abuse, and lost productivity, result in annual costs of $560 to $635 billion, which exceeds health care expenditures associated with heart disease and cancer combined.^1^

Chronic pain is also a pressing issue among US veterans, among whom the prevalence of this condition is documented to be several times higher than in the general population.^3^ Diagnoses of chronic pain, along with conditions like posttraumatic stress, anxiety, and substance abuse, have been found to be higher among individuals exposed to combat.^4^ This is important, as the US military recently completed the longest sustained period of combat exposure in its history (2001-2021).^5,6,7^ This timeframe also coincided with increased numbers of women entering uniformed service, and there are now more women combat veterans than at any other time in US history.^7^ The rigors of multiple deployments to combat zones have the potential to exert adverse effects on active-duty servicewomen (ADSW)^3,7,8,9^ as well as civilian women dependents whose spouse or partner served on active duty. This includes not just the potential for actual combat injury but secondary effects from psychological stress, anxiety, and emotional trauma.^3,8^ The impact of these repeated aspects of combat deployments on the development of chronic pain in women servicemembers and dependents has not been adequately studied.

In this context, we used data from the Military Health System (MHS) to examine the associations of service during a time period of heightened deployment intensity (eg, the most intense phases of the wars in Afghanistan and Iraq, 2006-2013)^5^ with the subsequent diagnosis of chronic pain among ADSW and women civilian dependents of active-duty personnel. Data from the MHS have been successfully used in the past to examine aspects of clinical care and health policy in relation to pain disorders and the opioid epidemic.^10,11,12^ We compared ADSW and women civilian dependents with service during 2006 to 2013 with like individuals serving at a time of reduced deployment intensity (2014-2020). We hypothesized that service in the period of heightened deployment intensity would be associated with an increased risk of a subsequent chronic pain diagnosis among ADSW compared with civilian dependents and servicewomen serving on active duty in 2014 to 2020.

## Methods

This study was deemed exempt by the Uniformed Services University of the Health Sciences and Partners institutional review boards with a waiver of informed consent because data were retrospectively reviewed and deidentified. The work was conducted in line with the Strengthening the Reporting of Observational Studies in Epidemiology (STROBE) reporting guideline.

This investigation used administrative and health care data (fiscal years 2006-2020) obtained from the MHS Data Repository. The means by which data were collected, prepared, made available for research, and accessed has been described in detail in prior publications.^10,12,13^ The study cohort included all adult (age 18-64 years) ADSW serving in the 4 constituent branches of the Department of Defense (Army, Air Force, Navy, and Marine Corps) at any time point in the period under study, as well as adult civilian women dependents of active-duty servicemembers serving in the same timeframe. Cohorts were established based on the time periods of 2006 to 2013 and 2014 to 2020, with the former timeframe indicative of heightened combat exposure and deployment intensity.^5^

Claims data of eligible individuals were surveyed for the diagnosis of a chronic pain condition using an *International Classification of Diseases, Ninth Revision *(*ICD-9*) and *International Statistical Classification of Diseases and Related Health Problems, Tenth Revision *(*ICD-10*) coding algorithm (eTable 1 in Supplement 1) aligned with previously published approaches.^11,14^ Eligible ADSW and dependents were surveyed from the initial medical encounter within our dataset to an end point of discharge from service or December 31, 2020. We were able to continue to observe ADSW who retired or were separated with a 30% disability or greater, irrespective of the site of service, as these individuals retain their military insurance benefit.^10,12,13^ Individuals who received a chronic pain diagnosis at any time over the surveillance period were considered in the chronic pain group. Individuals who did not receive a chronic pain diagnosis were included in the denominator population for both time periods.

We obtained sociodemographic and additional clinical data from the MHS Data Repository, including age (categorized as 18-24, 25-34, 35-44, 45-54, and ≥55 years), race (obtained by self-report as standard collection with enlistment and categorized as American Indian or Alaska Native, Asian or Pacific Islander, Black, White, and other), branch of service, census region, sponsor rank (junior enlisted, senior enlisted, warrant officer, junior officer, senior officer, other), presence of Charlson index comorbidities, and the presence of a comorbid mental health condition (eTable 2 in Supplement 1) based on prior literature that has suggested that these factors are associated with the development of chronic pain.^7,8,9,10^ Hispanic ethnicity was not available in our dataset. Among the active-duty cohort, we also surveyed for the development of the polytrauma clinical triad, as defined by Laughter et al^11^ and consisting of comorbid diagnoses of traumatic brain injury; chronic pain, sciatica, or backache; and posttraumatic stress.^11^

### Statistical Analysis

In all analyses, the diagnosis of chronic pain was considered the primary outcome and the interaction of active-duty status and time of service the primary exposure, with dependent women in 2014 to 2020 considered the reference group. As chronic pain is a disqualifying condition, it would not have been present at the time of enlistment. Initial, unadjusted, bivariate comparisons were made between variables using the χ^2^ test. We then used multivariable logistic regression to adjust for confounding using a term for active duty vs dependent, a term for the time period, and an interaction term. In adjusted analyses, we used reweighted estimating equations^15^ to handle missing race based on the recommendations of the *JAMA Surgery* guide for military claims data.^13^ Secondarily, and in line with previous investigations,^10,12,16^ we used sponsor rank as a proxy for socioeconomic status and assessed the interactions among this variable, ADSW status, and time of service. In all these analyses, senior officers were used as the reference group and junior enlisted status was considered representative of lower socioeconomic strata, based on prior work indicating those with junior enlisted sponsor rank have attitudes toward health care use, risk behaviors, and medical outcomes similar to civilian counterparts of lower socioeconomic status.^10,12,16^ As the development of chronic pain in this population is postulated to exist at the intersection of baseline risk, sociodemographic characteristics, and combat exposure,^3,8,9,17^ we addressed this by assessing for an interaction between sponsor rank and branch of service, using senior officer sponsor rank in the Air Force as the reference group. Here, enlisted ranks in the Army or Marine Corps were considered indicative of more intense combat exposure.^5,6,7^

The results of all analyses are presented using odds ratios (ORs) and 95% CIs with 2-sided *P* values. Statistical significance was established for variables with OR and 95% CI exclusive of 1.0 and *P* < .05. All analyses were performed using SAS software version 9.4 (SAS Institute). Data were analyzed from September 2023 to April 2024.

## Results

We included 3 473 401 individuals (median [IQR] age, 29.0 [22.0-46.0] years; 1 200 787 [34.6%] aged 18-24 years; 644 478 ADSW [18.6%]). Data collection was complete for all variables except race and Census region (Table 1). Among 1 087 899 individuals who self-reported race, the population consisted of 16 532 American Indian or Alaska Native individuals (1.5%), 83 910 Asian or Pacific Islander individuals (7.7%), 265 451 Black individuals (24.4%), 658 236 White individuals (60.5%), and 63 770 individuals who identified as other race (5.9%).

**Table 1.** Unadjusted Baseline Sociodemographic and Clinical Characteristics of the Study Cohort

Characteristic	Population, No. (%)		*P* values^a^
	Total (N = 3 473 401)	With chronic pain (n = 324 499)	
Age group, y			
18-24	1 200 787(34.6)	49 290(15.2)	<.001
25-34	880 443(25.3)	73 840(22.8)	
35-44	461 234(13.3)	65 510(20.2)	
45-54	339 857(9.8)	65 189(20.1)	
≥55	591 080(17.0)	70 670(21.8)	
Race and ethnicity			
American Indian or Alaska Native	16 532(0.5)	1231(0.4)	<.001
Asian or Pacific Islander	83 910(2.4)	7986(2.5)	
Black	265 451(7.6)	37 441(11.5)	
White	658 236(19.0)	89 629(27.6)	
Other^b^	63 770(1.8)	20 339(6.3)	
Missing	2 385 502(68.7)	167 873(51.7)	
Sponsor status			
Active	644 478 (18.6)	76 274 (23.5)	<.001
Dependent	2 828 923 (81.4)	248 225 (76.5)	
Time cohort			
2006-2013	2 281 546 (65.7)	271 438 (83.6)	<.001
2014-2020	1 191 855 (34.3)	53 061 (16.4)	
Exposure variable			
2006-2013 ADSW	394 429 (11.4)	58 484 (18.0)	<0.001
2014-2020 ADSW	250 049 (7.2)	17 790 (5.5)	
2006-2013 dependent	1 887 117 (54.3)	212 954 (65.6)	
2014-2020 dependent	941 806 (27.1)	35 271 (10.9)	
Service			
Army	1 420 742 (40.9)	160 591 (49.5)	<.001
Air Force	904 093 (26.0)	80 037 (24.7)	
Navy	794 325 (22.9)	59780 (18.4)	
Marine Corps	354 241 (10.2)	24 091 (7.4)	
Rank			
Senior enlisted	1 796 251 (51.7)	206 836 (63.7)	<.001
Senior officer	211 591 (6.1)	19 068 (5.9)	
Junior officer	343 543 (9.9)	25 224 (7.8)	
Junior enlisted	1 043 023 (30.0)	64 670 (19.9)	
Warrant officer	70 719 (2.0)	8521 (2.6)	
Other	8274 (0.2)	178 (0.1)	
Census region			
Midwest	282 198 (8.1)	27 774 (8.6)	<.001
Northeast	131 197 (3.8)	12 302 (3.8)	
South	1 634 400 (47.1)	169 215 (52.1)	
West	987 102(28.4)	82 909 (25.5)	
Other	359 159 (10.3)	30 303 (9.3)	
Missing	79 345 (2.3)	1996 (0.6)	
Mental illness			
Yes	2 323 857 (66.9)	154 400 (47.6)	<.001
No	1 149 544 (33.1)	170 099 (52.4)	
Comorbidities			
Myocardial infraction			
Yes	27 916 (0.8)	6419 (2.0)	<.001
No	3 445 485 (99.2)	318 080 (98.0)	
Congestive heart failure			
Yes	56 092 (1.6)	12 291 (3.8)	<.001
No	3 417 309 (98.4)	312 208 (96.2)	
Peripheral vascular disease			
Yes	96 008 (2.8)	20 807 (6.4)	<.001
No	3,77 393 (97.2)	303 692 (93.6)	
Cerebrovascular disease			
Yes	119 849 (3.5)	25 145 (7.7)	<.001
No	3 353 552 (96.5)	299 354 (92.3)	
Dementia			
Yes	6905 (0.2)	1386 (0.4)	<.001
No	3 466 496 (99.8)	323 113 (99.6)	
Chronic pulmonary disease			
Yes	704 785 (20.3)	114 782 (35.4)	<.001
No	2 768 616 (79.7)	209 717 (64.6)	
Connective tissue disease or rheumatic disease			
Yes	93 263 (2.7)	24 299 (7.5)	<.001
No	3 380 138 (97.3)	300 200 (92.5)	
Peptic ulcer disease			
Yes	52 853 (1.5)	13 099 (4.0)	<.001
No	3 420 548 (98.5)	311 400 (96.0)	
Mild liver disease			
Yes	178 781 (5.1)	36 317 (11.2)	<.001
No	3 294 620 (94.9)	288 182 (88.2)	
Diabetes without complication			
Yes	275 002 (7.9)	47 016 (14.5)	<.001
No	3 198 399 (92.1)	277 483 (85.5)	
Diabetes with complication			
Yes	74 378 (2.1)	14 781 (4.6)	<.001
No	3 399 023 (97.9)	309 718 (95.4)	
Paraplegia and hemiplegia			
Yes	21 656 (0.6)	5839 (1.8)	<.001
No	3 451 745 (99.4)	318 660 (98.2)	
Kidney disease			
Yes	63 061 (1.8)	12 252 (3.8)	<.001
No	3 410 340 (98.2)	312 247 (96.2)	
Any cancer			
Yes	173 578 (5.0)	28 096 (8.7)	<.001
No	3 299 823 (95.0)	296 403 (91.3)	
Moderate or severe liver disease			
Yes	7672 (0.2)	1712 (0.5)	<.001
No	3 465 729 (99.8)	322 787 (99.5)	
Metastatic carcinoma			
Yes	28 858 (0.8)	319 239 (98.4)	<.001
No	3 444 543 (99.2)	5260 (1.6)	
AIDS/HIV			
Yes	2637 (0.1)	404 (0.1)	<.001
No	3 470 764 (99.9)	324 095 (99.9)	

Abbreviation: ADSW, active-duty servicewomen.

^a^ Calculated using χ^2^.

^b^ Includes individuals who self-identified as other race.

We identified the diagnosis of chronic pain in 324 499 individuals (9.3%). The polytrauma clinical triad was identified in 2280 of 631 270 ADSW (0.3%). We identified chronic pain in 58 484 of 394 429 ADSW in service during 2006 to 2013 (14.8%) and in 212 954 of 1 887 117 dependents from this period (11.3%). Among 250 049 ADSW serving in 2014 to 2020, 17 790 had chronic pain (7.1%), while among 941 806 dependents in this time period, 35 271 (3.7%) had chronic pain. In adjusted analyses, compared with ADSW in 2014 to 2020, ADSW in 2006 to 2013 had a 53% increase in the odds of chronic pain (OR, 1.53; 95% CI, 1.48-1.58) (Table 2). The odds of chronic pain among dependents in 2006 to 2013 was also significantly higher compared with dependents from 2014 to 2020 (OR, 1.96; 95% CI, 1.93-1.99). The odds of chronic pain were elevated among ADSW in 2014 to 2020 compared with dependents in this same time period (OR, 1.20; 95% CI, 1.19-1.24). In this model, both comorbid mental conditions (OR, 1.67; 95% CI, 1.65-1.69) and service in the Army or Marine Corps were associated with elevated odds of chronic pain (Table 2).

**Table 2.** Multivariable-Adjusted Logistic Regression Analysis of the Association of Time and Beneficiary Status With Chronic Pain^a^

Characteristic	Population, No. (%)		OR (95% CI)	*P* value
	Total	With chronic pain		
Age group, y				
18-24	346 123 (31.8)	28 515 (8.2)	0.53 (0.52-0.54)	<.001
25-34	346 090 (31.8)	44 637 (12.9)	0.68 (0.66-0.69)	<.001
35-44	195 422 (18.0)	37 081 (19.0)	0.93 (0.91-0.95)	<.001
45-54	110 661 (10.2)	26 587 (24.0)	1.26 (1.23-1.28)	<.001
≥55	89 603 (8.2)	19 806 (22.1)	1 [Reference]	NA
Race and ethnicity				
American Indian or Alaska Native	16 532 (1.5)	1231 (7.5)	0.49 (0.46-0.52)	<.001
Asian or Pacific Islander	83 910 (7.7)	7986 (9.5)	0.51 (0.50-0.52)	<.001
Black	265 451 (24.4)	37 441 (14.1)	0.76 (0.75-.077)	<.001
White	658 236 (60.5)	89 629 (13.6)	1 [Reference]	NA
Other^b^	63 770 (5.9)	20 339 (31.9)	3.46 (3.40-3.53)	<.001
Role				
Active Duty	631 270 (58.0)	73 047 (11.6)	1.06 (1.04-1.08)	<.001
Dependent	456 629 (42.0)	83 579 (18.3)	1 [Reference]	
Time cohort				
2006-2013	751 402 (69.1)	131 455 (17.5)	1.73 (1.70-1.76)	<.001
2014-2020	336 497 (30.9)	25 171 (7.5)	1 [Reference]	
Exposure variable				
2006-2013 ADSW	388 484 (35.7)	55 694 (14.3)	NA	NA
2014-2020 ADSW	242 786 (22.3)	17 353 (7.2)	NA	NA
2006-2013 Dependent	362 918 (33.4)	75 761 (20.9)	NA	NA
2014-2020 Dependent	93 711 (8.6)	7818 (8.3)	NA	NA
Interaction				
ADSW in 2006-2013 vs ADSW in 2014-2020	NA	NA	1.53 (1.48-1.58)	<.001
ADSW in 2006-2013 vs dependent in 2006-2013	NA	NA	0.94 (0.92-0.96)	<.001
ADSW in 2006-2013 vs dependent in 2014-2020	NA	NA	1.85 (1.81-1.89)	<.001
ADSW in 2014-2020 vs dependent in 2006-2013	NA	NA	0.61 (0.59-0.63)	<.001
ADSW in 2014-2020 vs dependent in 2014-2020	NA	NA	1.20 (1.16-1.24)	<.001
Dependent in 2006-2013 vs dependent in 2014-2020	NA	NA	1.96 (1.93-1.99)	<.001
Branch				
Army	446 193 (41.0)	89 071 (20.0)	1.94 (1.91-1.96)	<.001
Air Force	294 711 (27.1)	33 759 (11.5)	1 [Reference]	NA
Navy	253 659 (23.3)	23 901 (9.4)	1.14 (1.12-1.16)	<.001
Marine Corps	93 336 (8.6)	9895 (10.6)	1.28 (1.25-1.31)	<.001
Rank				
Senior enlisted	454 907 (41.8)	89 933 (19.8)	1.32 (1.29-1.35)	<.001
Senior officer	40 282 (3.7)	8132 (20.2)	1 [Reference]	NA
Junior officer	108 328 (10.0)	12 212 (11.3)	0.99 (0.96-1.02)	.67
Junior enlisted	463 008 (42.6)	42 307 (9.1)	1.49 (1.45-1.53)	<.001
Warrant officer	17 095 (1.6)	3907 (22.9)	1.04 (1.00-1.09)	.03
Other	4279 (0.4)	135 (3.2)	0.19 (0.15-0.24)	<.001
Census region				
Midwest	88 684 (8.2)	12 124 (13.7)	0.94 (0.92-0.96)	<.001
Northeast	34 744 (3.2)	5624 (16.2)	0.86 (0.83-.088)	<.001
South	471 848 (43.4)	82 137 (17.4)	1 [Reference]	NA
West	284 962 (26.2)	36 777 (12.9)	0.84 0(.83-0.85)	<.001
Other	136 685 (12.6)	18 168 (13.3)	0.88 (0.86-0.90)	<.001
Missing	70 976 (6.5)	1796 (2.5)	0.25 (0.23-.027)	<.001
Comorbidities				
Myocardial infraction	5388 (0.5)	1766 (32.8)	1.08 (1.02-1.13)	.003
Congestive heart failure	10 783 (1.0)	3406 (31.6)	1.17 (1.13-1.22)	<.001
Peripheral vascular disease	18 804 (1.7)	5924 (31.5)	1.19 (1.15-1.22)	<.001
Cerebrovascular disease	25 838 (2.4)	7598 (29.4)	1.13 (1.10-1.16)	<.001
Dementia	1370 (0.1)	369 (26.9)	0.88 (0.79-0.98)	.02
Chronic pulmonary disease	214 615 (19.7)	49 286 (23.0)	1.45 (1.43-1.47)	<.001
Connective tissue disease or rheumatic disease	23 305 (2.1)	8011 (34.4)	1.86 (1.81-1.91)	<.001
Peptic ulcer disease	14 404 (1.3)	4593 (32.0)	1.48 (1.43-1.54)	<.001
Mild liver disease	43 300 (4.0)	11 975 (27.7)	1.16 (1.14-1.19)	<.001
Diabetes without complication	58 640 (5.4)	15 889 (27.1)	1.16 (1.14-1.19)	<.001
Diabetes with complication	13 939 (1.3)	4499 (32.3)	1.15 (1.11-1.19)	<.001
Paraplegia and hemiplegia	5621 (0.5)	2040 (36.3)	1.82 (1.72-1.92)	<.001
Kidney disease	13 139 (1.2)	3806 (29.0)	1.09 (1.05-1.13)	<.001
Any cancer	40 932 (3.8)	10 202 (24.9)	1.12 (1.09-1.14)	<.001
Moderate or severe liver disease	1567 (0.6)	502 (32.0)	1.13 (1.03-1.24)	.006
Metastatic carcinoma	6549 (0.6)	1833 (28.0)	1.10 (1.04-1.16)	<.001
AIDS/HIV	727 (0.1)	157 (21.6)	0.93 (0.77-1.11)	.44
Mental illness	414 661 (38.1)	81 733 (19.7)	1.67 (1.65-1.69)	<.001

Abbreviations: ADSW, active-duty servicewomen; NA, not applicable; OR, odds ratio.

^a^ Analyses were conducted among the population with complete race and ethnicity data.

^b^ Includes individuals who self-identified as other race.

In the primary model, our proxy for socioeconomic status was also significantly associated with increased odds of chronic pain (junior enlisted ADSW and dependents: OR, 1.49; 95% CI, 1.45-1.53). This was reinforced in further interaction analysis, which found that junior enlisted ADSW in 2006 to 2013 had a 95% increase in the odds of chronic pain (OR, 1.95; 95% CI, 1.83-2.09) (Table 3), while junior enlisted dependents in 2006 to 2013 experienced more than a 3-fold increase in the odds of chronic pain (OR, 3.05; 95% CI, 2.87-3.25) compared with senior officers.

**Table 3.** Multivariable-Adjusted Logistic Regression Assessing Interactions of Time, Beneficiary Status, and Rank With Chronic Pain

Characteristic	Population, No. (%)		OR (95% CI)	*P* value
	Total	With chronic pain		
Junior enlisted				
2006-2013 ADSW	194 486 (17.9)	17 334 (8.9)	1.95 (1.83-2.09)	<.001
2006-2013 Dependent	45 395 (4.2)	7860 (17.3)	3.05 (2.87-3.25)	<.001
2014-2020 ADSW	196 801 (18.1)	14 291 (7.3)	1.90 (1.77-2.03)	<.001
2014-2020 Dependent	26 326 (2.4)	2822 (10.7)	2.03 (1.91-2.17)	<.001
Junior officer				
2006-2013 ADSW	43 261 (4.0)	4947 (11.4)	1.67 (1.54-1.81)	<.001
2006-2013 Dependent	31 363 (2.9)	5564 (17.7)	2.65 (2.12-2.41)	<.001
2014-2020 ADSW	23 557 (2.2)	1083 (4.6)	0.88 (0.77-0.99)	.04
2014-2020 Dependents	10 147 (0.9)	618 (6.1)	0.96 (0.89-1.03)	.29
Other				
2006-2013 ADSW	590 (0.1)	64 (10.9)	2.76 (1.85-4.11)	<.001
2006-2013 Dependent	23 (0.0)	NA^a^	<0.001 (<0.001->999.9)	.70
2014-2020 ADSW	3659 (0.3)	71 (1.9)	0.48 (0.35-0.65)	<.001
2014-2020 Dependent	NA^a^	NA^a^	<0.001 (<0.001->999.9)	.83
Senior enlisted				
2006-2013 ADSW	135 614 (12.5)	28 702 (21.2)	3.51 (3.29-3.74)	<.001
2006-2013 Dependent	250 970 (23.1)	55 479 (22.1)	2.77 (2.60-2.94)	<.001
2014-2020 ADSW	17 782 (1.6)	1865 (10.5)	2.00 (1.80-2.22)	<.001
2014-2020 Dependent	50 541 (4.6)	3887 (7.7)	1.15 (1.08-1.22)	<.001
Senior officer				
2006-2013 ADSW	11 261 (1.0)	3533 (31.4)	4.54 (4.13-4.99)	<.001
2006-2013 Dependent	23 767 (2.2)	4236 (17.8)	1.93 (1.80-2.06)	<.001
2014-2020 ADSW	699 (0.1)	33 (4.7)	0.74 (0.39-1.39)	.36
2014-2020 Dependent	4555 (0.4)	330 (7.2)	1 [Reference]	NA
Warrant officer				
2006-2013 ADSW	3272 (0.3)	1114 (34.1)	4.33 (3.73-5.02)	<.001
2006-2013 Dependent	11 400 (1.0)	2622 (23.0)	2.13 (1.98-2.29)	<.001
2014-2020 ADSW	288 (0.0)	NA^a^	0.46 (0.14-1.44)	.18
2014-2020 Dependent	2135 (0.2)	161 (7.5)	0.93 (0.83-1.05)	.26

Abbreviations: ADSW, active-duty servicewomen; NA not applicable or not available.

^a^ Data not available because cells with fewer than 10 individuals are censored.

In our secondary analysis relying on the interaction between sponsor rank and branch as a proxy for combat exposure, we found significant elevations in the odds of chronic pain among junior enlisted ADSW in the Army (OR, 2.69; 95% CI, 2.57-2.81) and Marine Corps (OR, 1.58; 95% CI, 1.49-1.67), as well as senior enlisted in both branches (Army: OR, 1.91; 95% CI, 1.83-1.99; Marine Corps: OR, 1.36; 95% CI, 1.30-1.44) (Table 4) compared with senior officers in the Air Force.

**Table 4.** Multivariable-Adjusted Logistic Regression Assessing Interactions of Rank and Service Branch With the Development of Chronic Pain

Characteristic	Population, No. (%)		OR (95% CI)	*P* value
	Total	With chronic pain		
Junior Enlisted				
Army	192 096 (17.7)	28 976 (15.1)	2.69 (2.57-2.81)	<.001
Navy	117 124 (10.8)	4747 (4.1)	0.94 (0.89-0.99)	.03
Marine Corps	49 226 (4.5)	3190 (6.5)	1.58 (1.49-1.67)	<.001
Air Force	104 562 (9.6)	5394 (5.2)	0.80 (0.76-0.84)	<.001
Senior Enlisted				
Army	178 593 (16.4)	45 589 (25.5)	1.91 (1.83-1.99)	<.001
Navy	102 244 (9.4)	15 766 (15.41)	1.30 (1.24-1.36)	<.001
Marine Corps	33 736 (3.1)	5531 (16.4)	1.36 (1.30-1.44)	<.001
Air Force	140 334 (12.9)	23 047 (16.4)	1.11 (1.06-1.16)	<.001
Junior Officer				
Army	42 224 (3.9)	6890 (16.3)	1.50 (1.42-1.58)	<.001
Navy	24 038 (2.2)	1833 (7.6)	0.94 (0.88-0.99)	.05
Marine Corps	6643 (0.6)	563 (8.5)	0.79 (0.73-0.87)	<.001
Air Force	35 423 (3.3)	2926 (8.3)	0.84 (0.79-0.89)	<.001
Senior Officer				
Army	17 406 (1.6)	4218 (24.2)	1.29 (1.22-1.36)	<.001
Navy	7430 (0.7)	1229 (16.5)	1.06 (0.99-1.13)	.07
Marine Corps	1997 (0.2)	314 (15.7)	0.92 (0.83-1.02)	.13
Air Force	13 449 (1.2)	2371 (17.6)	1 [Reference]	NA
Warrant Officer				
Army	13 750 (1.3)	3285 (23.9)	1.52 (1.43-1.61)	<.001
Navy	1604 (0.1)	321 (20.0)	1.36 (1.22-1.52)	<.001
Marine Corps	1726 (0.2)	296 (17.1)	1.18 (1.04-1.34)	.01
Air Force	15 (0.0)	NA^a^	11.67 (3.48-39.10)	<.001
Other				
Army	2124 (0.2)	113 (5.3)	0.47 (0.36-.062)	<.001
Navy	1219 (0.1)	NA^a^	0.02 (0.00-0.07)	<.001
Marine Corps	NA^a^	NA^a^	0.03 (0.00-0.80)	.04
Air Force	928 (0.1)	16 (1.7)	0.09 (0.04-0.20)	<.001

Abbreviations: NA not applicable or not available; OR, odds ratio.

^a^ Data not available because cells with fewer than 10 individuals are censored.

ADSW serving in 2006 to 2013 with chronic pain were significantly more likely to be diagnosed with the polytrauma clinical triad compared with ADSW with no chronic pain from 2014 to 2020 (OR, 7.63; 95% CI, 6.49-8.98) (Table 5). ADSW with chronic pain from 2006 to 2013 were also significantly more likely to be diagnosed with the polytrauma clinical triad than ADSW with chronic pain serving in 2014 to 2020 (OR, 1.69; 95% CI, 1.38-2.07).

**Table 5.** Results of the Multivariable-Adjusted Logistic Regression Assessing for Associations With the Development of PCT Among ADSW

Variable	Population, No. (%)		OR (95% CI)	*P* value
	Total (n = 631 270 [100])	With PCT (n = 2280 [0.3])		
Time cohort				
2006-2013	388 484 (61.5)	1952 (0.5)	1.75 (1.53-2.00)	<.001
2014-2020	242 786 (38.5)	328 (0.1)	1 [Reference]	NA
Chronic pain	73 047 (11.6)	1139 (1.3)	4.36 (3.84-4.94)	<.001
Interaction				
ADSW in 2006-2013 with chronic pain vs ADSW in 2014-2020 with chronic pain	NA	NA	1.69 (1.38-2.07)	<.001
ADSW in 2006-2013 with chronic pain vs ADSW in 2006-2013 with no chronic pain	NA	NA	4.21 (3.83-4.64)	<.001
ADSW in 2006-2013 with chronic pain vs ADSW in 2014-2020 with no chronic pain	NA	NA	7.63 (6.49-8.98)	<.001
ADSW in 2014-2020 with chronic pain vs ADSW in 2006-2013 with no chronic pain	NA	NA	2.49 (2.03-3.04)	<.001
ADSW in 2014-2020 with chronic pain vs ADSW in 2014-2020 with no chronic pain	NA	NA	4.51 (3.58-5.68)	<.001
ADSW in 2006-2013 with no chronic pain vs ADSW in 2014-2020 with no chronic pain	NA	NA	1.81 (1.55-2.11)	<.001

Abbreviations: ADSW, active-duty servicewomen; NA not applicable; OR, odds ratio; PCT, polytrauma clinical triad.

## Discussion

This cohort study represents the first work to longitudinally examine chronic pain development in a population of US women, to our knowledge. Our study cohort includes ADSW routinely exposed to combat environments and the rigors of war-related deployments, as well as the women civilian dependents of individuals on active duty. This effort is advantaged by the larger number of individuals considered, as well as the ability to longitudinally survey for the development of chronic pain irrespective of the time of onset, or the environment of care in which the diagnosis was made. Further, the sociodemographic, educational, and vocational diversity of the modern US armed forces^10,11,12,13^ allowed us to assess for intersectionality among socioeconomic status, proxies for combat exposure, and the development of chronic pain in ways that smaller studies with more restricted populations are otherwise unable to achieve. Furthermore, these same characteristics of our cohort enable the translation of our findings to the working-age population of the US as a whole, as previous work has found that the composition of individuals covered by the MHS are comparable with the US population aged 18 to 64 years.^10,12,13^

The overall incidence of chronic pain in our cohort was 9.3%, which is comparable with prior estimates for chronic widespread pain among women as a whole^1^ and the incidence of chronic pain reported in the Health and Retirement Study.^18^ Our findings that comorbid mental health conditions were associated with an increased likelihood of chronic pain are also supported by previous investigations among purely civilian cohorts.^1,19^ We believe these facts reinforce the face validity of our results and the capacity for their translation to the broader population of US women, outside of the immediate implications they may hold for the MHS.

While the predilection for chronic pain disorders among US veterans and others exposed to combat has been widely recognized in the literature^3,4,9^ we believe that our investigation presents several novel paradigms. First, our study suggests a greater adverse impact from heightened deployment schedules, given the statistical difference between the rates of chronic pain development between ADSW serving in 2006 to 2013 compared with ADSW in 2014 to 2020. That some of this difference is directly due to exposure to combat and related trauma is reinforced by the significant association between the polytrauma clinical triad and ADSW with chronic pain who were serving in 2006 to 2013. However, the fact that the likelihood of chronic pain development was also significantly elevated among civilian dependent women from this same time period attests to the fact that the risk of chronic pain does not derive solely from combat exposure and the operational environment. The finding that civilian women dependents from 2006 to 2013 were at an even higher odds of chronic pain than ADSW in this time frame was particularly surprising to us and indicates that the inimical effects of heightened deployment cycles extend well beyond the active-duty individual to include stressors, such as fear and worry for the safety of the deployed spouse or partner, assuming duties of a single parent, income alterations, and disruptions in social support.

Our results also demonstrate the disparate impact that combat exposure has based on socioeconomic status. In the main analysis, we found a significant increase in the odds of a chronic pain diagnosis among junior enlisted sponsor rank ADSW, our proxy for socioeconomic status. The association with combat exposure was evident in secondary testing, where we considered the interaction between time of service and the polytrauma clinical triad in a cohort restricted to ADSW. Here, ADSW from 2006 to 2013 with chronic pain were at significantly higher odds of receiving a diagnosis of the polytrauma clinical triad, with a more than 7-fold increase in likelihood compared with ADSW from 2014 to 2020 without chronic pain.

The development of chronic pain following military service has been postulated to result from a combination of background predilection, exposure to trauma, and the effects of comorbid medical and behavioral conditions that may have been preexisting or developed as direct sequelae of the traumatic event.^3,4,9^ The potential for higher rates of chronic pain in women veterans has been theorized to result from differences in support structures, family conflict, coping strategies, stress regulation, and exposure to military sexual trauma.^3,8^ Our results suggest that these contributing factors may carry over to the women dependents of combat veterans in addition, indicating a line of research that requires urgent further exploration.

At a minimum, our results hold immediate meaning for the MHS, Department of Veterans Affairs, and civilian health care systems that provide services for large numbers of civilian dependents of military servicemembers. The results regarding the associations of combat exposure and operational intensity with chronic pain development can be used by stakeholders in the future for resource allocation and deployment of support services that could mitigate risk. In light of our other findings, such approaches could be specifically targeted to individuals at greatest risk of chronic pain development, including those from lower socioeconomic strata or individuals with behavioral health conditions. We would also stress the importance of offering these services to civilian dependents of servicemembers who have these characteristics or who possess such risk factors themselves. Due to the representative nature of the MHS population in relation to the US national population, we believe that these recommendations could also apply to civilian women who are exposed to firearm injuries or mass casualty events as well as the spouses or partners of individuals who experience such types of trauma.

### Limitations

This study has some limitations. We were limited by our reliance on claims-based data, with inherent issues in terms of coding accuracy and limited clinical granularity. As a result, we cannot be certain of the specific diagnostic criteria that were used to support determinations of chronic pain or any of the other conditions documented within the MHS claims. If these or other temporal factors changed in the periods under study, there could be an impact on our findings. However, we are unaware of large-scale systemic differences in the MHS in terms of diagnosis and management of chronic pain in the time periods under study. There were issues around reporting of race, which were handled to the best of our ability using validated statistical methods. We are also reliant on the use of time periods as proxies for exposure to the combat environment and operational intensity. There were reductions in the population over time due to downsizing of the military. Changes in the population at risk were addressed in our analytic approach. We do not possess data on deployment locations or length, type of combat experienced, or the nature of specific injuries sustained as a result of combat. While there may be prospect for some confounding in this regard, we would emphasize that our secondary testing around the associations of chronic pain with service branch and the polytrauma clinical triad all reinforce our postulates regarding the association of the combat environment with the development of this condition. Although our ability to capture longitudinal data and remote diagnoses exceeds that of many other retrospective investigations, we are still restricted to data captured by the MHS in association with the delivery of health care. Individuals with chronic pain who did not report symptoms, or those who may have been diagnosed after separation from service, or once receiving care through the VA would not be identified in the datasets we used. Therefore, we do acknowledge that the prevalence of chronic pain in our population is likely underestimated.

## Conclusions

This cohort study represents one of the largest and most comprehensive longitudinal assessments of the diagnosis of chronic pain in ADSW and civilian dependents. We found strong and pervasive signals for the association of combat exposure with the development of chronic pain in active-duty personnel and civilian dependents. The likelihood of chronic pain was further increased in the setting of lower socioeconomic status and mental health conditions. More intentional resource allocation and preventative services targeted to servicemembers and civilian dependents with these characteristics may address a potential missed opportunity to reduce the risk of chronic pain development in at-risk individuals. In light of the representative nature of the population served by the MHS, these findings may apply to the civilian health sector as well.

## Notes

Supplement 1. **eTable 1.** List of Chronic Pain Conditions and Associated ICD-9 and 10 Codes **eTable 2.** List of Mental Health Conditions and Associated ICD-9 and 10 Codes

Supplement 2.
Data Sharing Statement

## References

[1] Andrews P, Steultjens M, Riskowski J. Chronic widespread pain prevalence in the general population: a systematic review. Eur J Pain. 2018;22(1):5-18. doi:10.1002/ejp.1090 28815801

[2] Institute of Medicine. Relieving Pain in America: A Blueprint for Transforming Prevention, Care, Education and Research. The National Academies Press; 2011. 22553896

[3] Driscoll MA, Higgins DM, Seng EK, . Trauma, social support, family conflict, and chronic pain in recent service veterans: does gender matter? Pain Med. 2015;16(6):1101-1111. doi:10.1111/pme.12744 25930005

[4] Thomas MM, Harpaz-Rotem I, Tsai J, Southwick SM, Pietrzak RH. Mental and physical health conditions in US combat veterans: results from the National Health and Resilience in Veterans Study. Prim Care Companion CNS Disord. 2017;19(3). doi:10.4088/PCC.17m02118 28657698

[5] Belmont PJ, Owens BD, Schoenfeld AJ. Musculoskeletal injuries in Iraq and Afghanistan: epidemiology and outcomes following a decade of war. J Am Acad Orthop Surg. 2016;24(6):341-348. doi:10.5435/JAAOS-D-15-00123 27115793

[6] Patzkowski JC, Rivera JC, Ficke JR, Wenke JC. The changing face of disability in the US Army: the Operation Enduring Freedom and Operation Iraqi Freedom effect. J Am Acad Orthop Surg. 2012;20(suppl 1):S23-S30. doi:10.5435/JAAOS-20-08-S23 22865131

[7] Rivera JC, Hylden CM, Johnson AE. Disability after deployment injury: are women and men service members different? Clin Orthop Relat Res. 2015;473(8):2448-2454. doi:10.1007/s11999-015-4180-6 25666145 PMC4488211

[8] Higgins DM, Kerns RD, Brandt CA, . Persistent pain and comorbidity among Operation Enduring Freedom/Operation Iraqi Freedom/Operation New Dawn veterans. Pain Med. 2014;15(5):782-790. doi:10.1111/pme.12388 24548466

[9] Weimer MB, Macey TA, Nicolaidis C, Dobscha SK, Duckart JP, Morasco BJ. Sex differences in the medical care of VA patients with chronic non-cancer pain. Pain Med. 2013;14(12):1839-1847. doi:10.1111/pme.12177 23802846 PMC3866355

[10] Chaudhary MA, Bhulani N, de Jager EC, . Development and validation of a bedside risk assessment for sustained prescription opioid use after surgery. JAMA Netw Open. 2019;2(7):e196673. doi:10.1001/jamanetworkopen.2019.6673 31290987 PMC6624809

[11] Laughter S, Khan M, Banaag A, Madsen C, Koehlmoos TP. Prevalence of polytrauma clinical triad among active duty service members. Mil Med. 2022;187(7-8):e856-e861. doi:10.1093/milmed/usab199 34050366

[12] Scully RE, Schoenfeld AJ, Jiang W, . Defining optimal length of opioid pain medication prescription after common surgical procedures. JAMA Surg. 2018;153(1):37-43. doi:10.1001/jamasurg.2017.3132 28973092 PMC5833616

[13] Schoenfeld AJ, Kaji AH, Haider AH. Practical guide to surgical data sets: military health system Tricare encounter data. JAMA Surg. 2018;153(7):679-680. doi:10.1001/jamasurg.2018.0480 29617526

[14] Smith HJ, Taubman SB, Clark LL. A burden and prevalence analysis of chronic pain by distinct case definitions among active duty U.S. military service members, 2018. Pain Physician. 2020;23(5):E429-E440. doi:10.36076/ppj.2020/23/E429 32967387

[15] Henry AJ, Hevelone ND, Lipsitz S, Nguyen LL. Comparative methods for handling missing data in large databases. J Vasc Surg. 2013;58(5):1353-1359.e6. doi:10.1016/j.jvs.2013.05.008 23830314

[16] Nurutdinova D, Chrusciel T, Zeringue A, . Mental health disorders and the risk of AIDS-defining illness and death in HIV-infected veterans. AIDS. 2012;26(2):229-234. doi:10.1097/QAD.0b013e32834e1404 22089375

[17] Jay MA, Bendayan R, Cooper R, Muthuri SG. Lifetime socioeconomic circumstances and chronic pain in later adulthood: findings from a British birth cohort study. BMJ Open. 2019;9(3):e024250. doi:10.1136/bmjopen-2018-024250 30850405 PMC6429846

[18] Janevic MR, McLaughlin SJ, Heapy AA, Thacker C, Piette JD. Racial and socioeconomic disparities in disabling chronic pain: findings from the Health and Retirement Study. J Pain. 2017;18(12):1459-1467. doi:10.1016/j.jpain.2017.07.005 28760648 PMC5682226

[19] Mundal I, Gråwe RW, Bjørngaard JH, Linaker OM, Fors EA. Prevalence and long-term predictors of persistent chronic widespread pain in the general population in an 11-year prospective study: the HUNT study. BMC Musculoskelet Disord. 2014;15:213. doi:10.1186/1471-2474-15-213 24951013 PMC4089927

