# Supplementary material for: Development of Chronic Pain Conditions Among Women in the Military Health System
Source: JAMA Netw Open. 2024 Jul 5;7(7):e2420393. doi: 10.1001/jamanetworkopen.2024.20393 (PMC11227075; doi:10.1001/jamanetworkopen.2024.20393)
Supplement: Supplement 1. — eTable 1. List of Chronic Pain Conditions and Associated ICD-9 and 10 Codes eTable 2. List of Mental Health Conditions and Associated ICD-9 and 10 Codes [file jamanetwopen-e2420393-s001.pdf]

Supplemental Online Content

Schoenfeld AJ, Cirillo MN, Gong J, et al. Development of chronic pain conditions among women in the military health system. *JAMA Netw Open*. 2024;7(7):e2420393. doi:10.1001/jamanetworkopen.2024.20393

eTable 1. List of Chronic Pain Conditions and Associated *ICD-9* and *ICD-10* Codes

eTable 2. List of Mental Health Conditions and Associated *ICD-9* and *ICD-10* Codes

This supplemental material has been provided by the authors to give readers additional information about their work.

© 2024 Schoenfeld AJ et al. *JAMA Network Open.*

Supplemental Table 1: List of chronic pain conditions and associated ICD-9 and 10 codes used in this investigation

ICD-10 Code	Chronic pain condition
G894	Chronic pain syndrome
G8921	Chronic pain due to trauma
G8922	Chronic post-thoracotomy pain
G8928	Other chronic postprocedural pain
G8929	Other chronic pain
M2550	Pain in unspecified joint
M25511	Pain in right shoulder
M25512	Pain in left shoulder
M25519	Pain in unspecified shoulder
M25521	Pain in right elbow
M25522	Pain in left elbow
M25529	Pain in unspecified elbow
M25531	Pain in right wrist
M25532	Pain in left wrist
M25539	Pain in unspecified wrist
M25541	Pain in joints of right hand
M25542	Pain in joints of left hand
M25549	Pain in joints of unspecified hand
M25551	Pain in right hip
M25552	Pain in left hip
M25559	Pain in unspecified hip
M25561	Pain in right knee
M25562	Pain in left knee
M25569	Pain in unspecified knee
M25571	Pain in right ankle and joints of right foot
M25572	Pain in left ankle and joints of left foot
M25579	Pain in unspecified ankle and joints of unspecified foot
M2559	Pain in other specified joint
M5410	Radiculopathy, site unspecified
M5411	Radiculopathy, occipito-atlanto-axial region
M5412	Radiculopathy, cervical region
M5413	Radiculopathy, cervicothoracic region
M5414	Radiculopathy, thoracic region
M5415	Radiculopathy, thoracolumbar region
M5416	Radiculopathy, lumbar region
M5417	Radiculopathy, lumbosacral region
M5418	Radiculopathy, sacral and sacrococcygeal region
M542	Cervicalgia
M5430	Sciatica, unspecified side

© 2024 Schoenfeld AJ et al. *JAMA Network Open.*

M5431	Sciatica, right side
M5432	Sciatica, left side
M5440	Lumbago with sciatica, unspecified side
M5441	Lumbago with sciatica, right side
M5442	Lumbago with sciatica, left side
M5450	Low back pain, unspecified
M5451	Vertebrogenic low back pain
M5459	Other low back pain
M546	Pain in thoracic spine
M5481	Occipital neuralgia
M5489	Other dorsalgia
M549	Dorsalgia, unspecified
M7910	Myalgia, unspecified site
M7911	Myalgia of mastication muscle
M7912	Myalgia of auxiliary muscles, head and neck
M7918	Myalgia, other site
M792	Neuralgia and neuritis, unspecified
M79601	Pain in right arm
M79602	Pain in left arm
M79603	Pain in arm, unspecified
M79604	Pain in right leg
M79605	Pain in left leg
M79606	Pain in leg, unspecified
M79609	Pain in unspecified limb
M79621	Pain in right upper arm
M79622	Pain in left upper arm
M79629	Pain in unspecified upper arm
M79631	Pain in right forearm
M79632	Pain in left forearm
M79639	Pain in unspecified forearm
M79641	Pain in right hand
M79642	Pain in left hand
M79643	Pain in unspecified hand
M79644	Pain in right finger(s)
M79645	Pain in left finger(s)
M79646	Pain in unspecified finger(s)
M79651	Pain in right thigh
M79652	Pain in left thigh
M79659	Pain in unspecified thigh
M79661	Pain in right lower leg
M79662	Pain in left lower leg
M79669	Pain in unspecified lower leg
M79671	Pain in right foot

© 2024 Schoenfeld AJ et al. *JAMA Network Open.*

M79672	Pain in left foot
M79673	Pain in unspecified foot
M79674	Pain in right toe(s)
M79675	Pain in left toe(s)
M79676	Pain in unspecified toe(s)
R070	Pain in throat
R071	Chest pain on breathing
R072	Precordial pain
R0781	Pleurodynia
R0782	Intercostal pain
R0789	Other chest pain
R079	Chest pain, unspecified
R1010	Upper abdominal pain, unspecified
R1011	Right upper quadrant pain
R1012	Left upper quadrant pain
R1013	Epigastric pain
R102	Pelvic and perineal pain
R1030	Lower abdominal pain, unspecified
R1031	Right lower quadrant pain
R1032	Left lower quadrant pain
R1033	Periumbilical pain
R1084	Generalized abdominal pain
R109	Unspecified abdominal pain
R521	Chronic intractable pain
R522	Other chronic pain
R529	Pain, unspecified
G501	Atypical facial pain
H5710	Ocular pain, unspecified eye
H5711	Ocular pain, right eye
H5712	Ocular pain, left eye
H5713	Ocular pain, bilateral
K146	Glossodynia
R6884	Jaw pain

© 2024 Schoenfeld AJ et al. *JAMA Network Open.*

ICD-9 Code	Chronic Pain Condition
3384	Chronic pain syndrome
33821	Chronic pain due to trauma
33822	Chronic post-thoracotomy pain
33828	Other chronic postoperative pain
33829	Other chronic pain
71940	Pain in joint, site unspecified
71941	Pain in joint, shoulder region
71942	Pain in joint, upper arm
71943	Pain in joint, forearm
71944	Pain in joint, hand
71945	Pain in joint, pelvic region and thigh
71946	Pain in joint, lower leg
71947	Pain in joint, ankle and foot
71948	Pain in joint, other specified sites
71949	Pain in joint, multiple sites
7231	Cervicalgia
7234	Brachial neuritis or radiculitis NOS
7241	Pain in thoracic spine
7242	Lumbago
7243	Sciatica
7245	Backache, unspecified
7244	Thoracic or lumbosacral neuritis or radiculitis, unspecified
7291	Myalgia and myositis, unspecified
7292	Neuralgia, neuritis, and radiculitis, unspecified
7295	Pain in limb
78096	Generalized pain
78650	Chest pain, unspecified
78651	Precordial pain
78652	Painful respiration
78659	Other chest pain
78900	Abdominal pain, unspecified site
78901	Abdominal pain, right upper quadrant
78902	Abdominal pain, left upper quadrant
78903	Abdominal pain, right lower quadrant
78904	Abdominal pain, left lower quadrant
78905	Abdominal pain, periumbilic
78906	Abdominal pain, epigastric
78907	Abdominal pain, generalized
78909	Abdominal pain, other specified site
7840	Headache
7841	Throat pain

© 2024 Schoenfeld AJ et al. *JAMA Network Open.*

3502	Atypical face pain
37991	Pain in or around eye
5296	Glossodynia
78492	Jaw pain

© 2024 Schoenfeld AJ et al. *JAMA Network Open.*

Supplemental Table 2: List of mental health conditions and associated ICD-9 and 10 codes used in this investigation

Mental Health Condition	ICD-10 Codes
**Anxiety disorders**	F40.00, F40.01, F40.02, F40.10, F40.11, F40.210, F40.218, F40.220, F40.228, F40.230, F40.231, F40.232 , F40.233, F40.240, F40.241, F40.242, F40.243, F40.248, F40.290, F40.291, F40.298, F40.8, F40.9, F41.0, F41.1, F41.3, F41.8, F41.9, F42.2, F42.3, F42.4, F42.8, F42.9
**Depressive disorders**	F32.1, F32.2, F32.3, F32.4, F32.5, F32.81, F32.89, F32.9, F32.A, F33.0, F33.1, F33.2, F33.3, F33.40, F33.41, F33.42, F33.8, F33.9, F34.1, F34.89, F34.9
**Adjustment disorders**	F43.2, F43.21, F43.22, F43.23, F43.24, F43.25, F43.29
**PTSD**	F43.10 F43.11, F43.12
**Severe mental illness**	F20.0, F20.1, F20.2, F20.3, F20.5, F20.8, F20.9, F22.0, F23.0, F24.0,F25.0, F25.1, F25.9, F28.0, F29.0, F30.1, F30.2, F30.23, F30.4, F30.8, F30.9, F31.0, F31.1, F31.2, F31.3, F31.4, F31.5, F31.6, F31.7, F31.8, F31.9,F34.0, F34.1, F34.8, F34.9

Mental Health Condition	ICD -9 Codes
**Anxiety disorders**	300.02, 300.01, 300.02, 300.09, 300.20-300.29, 300.3
**Depression**	296.21, 296.22, 296.23, 296.24, 296.25, 296.26, 296.20, 296.31, 296.32, 296.33, 296.34, 296.99, 296.30, 300.4, 301.12
**Adjustment disorders**	309.9, 309.0, 309.1, 309.24, 309.28, 309.3, 309.29, 309.4
**PTSD**	309.81
**Severe mental illness**	
**Schizophrenia**	295.30, 295.10, 295.20, 295.90, 295.60,
**Delusional disorders**	297.0, 297.1,297.2
**Brief psychotic disorder**	298.3-298.4,298.8
**Shared psychotic disorder**	297.3
**Schizoaffective disorders**	295.70
**Other psychotic disorder not due to a substance or known physiological condition**	298.9
**Unspecified psychosis**	298.9
**Manic episode**	296.04, 296.05, 296.06, 296.81, 296.00
**Bipolar disorder**	296.40, 296.44, 296.53, 296.54, 296.80
Cyclothymic disorder	301.10, 301.13, 301.12, 300.4, 296.99,

© 2024 Schoenfeld AJ et al. *JAMA Network Open.*
