# Supplementary material for: Development of Chronic Pain Conditions Among Women in the Military Health System
Source: JAMA Netw Open. 2024 Jul 5;7(7):e2420393. doi: 10.1001/jamanetworkopen.2024.20393 (PMC11227075; doi:10.1001/jamanetworkopen.2024.20393)
Supplement: Supplement 2. — Data Sharing Statement [file jamanetwopen-e2420393-s002.pdf]

Data Sharing Statement

Schoenfeld. Development of Chronic Pain Conditions Among Women in the Military Health System. *JAMA Netw Open*. Published July 05, 2024.
doi:10.1001/jamanetworkopen.2024.20393

Data
Data available: No

Additional Information
Explanation for why data not available: This is proprietary data of the Department of Defense and cannot be made available for public use by DSA
